# Application of AAPM TG 119 to volumetric arc therapy (VMAT)

**DOI:** 10.1120/jacmp.v13i5.3382

**Published:** 2012-09-06

**Authors:** Dinesh Kumar Mynampati, Ravindra Yaparpalvi, Linda Hong, Hsiang‐Chi Kuo, Dennis Mah

**Affiliations:** ^1^ Department of Radiation Oncology Montefiore Medical Center Bronx New York USA

**Keywords:** VMAT, IMRT, TG 119, quality assurance

## Abstract

The purpose of this study was to create AAPM TG 119 benchmark plans for volumetric arc therapy (VMAT) and to compare VMAT plans with IMRT plan data. AAPM TG 119 proposes a set of test clinical cases for testing the accuracy of IMRT planning and delivery system. For these test cases, we generated two treatment plans, the first plan using 7–9 static dMLC IMRT fields and a second plan utilizing one‐ or two‐arc VMAT technique. Dose optimization and calculations performed using 6 MV photons and Eclipse treatment planning system. Dose prescription and planning objectives were set according to the TG 119 goals. Plans were scored based on TG 119 planning objectives. Treatment plans were compared using conformity index (CI) for reference dose and homogeneity index (HI) (for $D_{5}‐D_{95}$). F or test cases prostate, head‐and‐neck, C‐shape and multitarget prescription dose are 75.6 Gy, 50.4 Gy, 50 Gy and 50 Gy, respectively. VMAT dose distributions were comparable to dMLC IMRT plans. Our planning results matched TG 119 planning results. For treatment plans studied, conformity indices ranged from 1.05–1.23 (IMRT) and 1.04–1.23 (VMAT). Homogeneity indices ranged from 4.6%–11.0% (IMRT) and 4.6%–10.5% (VMAT). The ratio of total monitor units necessary for dMLC IMRT to that of VMAT was in the range of 1.1–2.0. AAPM TG 119 test cases are useful to generate VMAT benchmark plans. At preclinical implementation stage, plan comparison of VMAT and IMRT plans of AAPM TG 119 test case allowed us to understand basic capabilities of VMAT technique.

PACS number: 87.55.Qr

## I. INTRODUCTION

The advantage of nonuniform beams is to deliver highly conformal distributions to target, while sparing organs at risk has led to improvements in clinical outcomes. Radiation beam modifiers to produce nonuniform beams are evolving continually by incorporating available technological advancements. Since inception, intensity‐modulated radiation therapy (IMRT) using multileaf collimators (MLC)^(1)^ has become widespread with a variety of different dose delivery methods (i.e. step‐and‐shoot, dynamic MLC, tomotherapy. The concept of volumetric modulated arc therapy (VMAT) was first proposed in 1995.^(2)^ More recently the work of Otto^(3)^ has led to the development of a commercial approach of VMAT called RapidArc (Varian Medical Systems, Palo Alto, CA). The architecture of this technique provides more number of degrees of freedom to optimize the dose delivery. Clinical advantages and comparison with present techniques for different sites have been reported.^4^, ^6^ To commission and QA the delivery system, Ling et al.^(7)^ proposed benchmark tests based upon the principles addressed by LoSasso et al.^(8)^ for dynamic IMRT QA. A guidance document^(9)^ for IMRT commissioning and guidelines for IMRT commissioning have recently been published^(10)^ by AAPM Task Group (TG) 119. The guidelines established test cases to benchmark the overall accuracy of IMRT planning and delivery. Specifically, AAPM TG 119 surveyed 10 institutions and applied simplified target structures with organs at risk (OAR) and planning goals using a variety of different delivery techniques, devices, and planning systems. In this report, we provide our experience applying AAPM, TG 119 to VMAT technology.

The goal of our work is to determine if VMAT is capable of delivering plans of comparable quality compared to IMRT plans using TG 119 as a metric.

## II. MATERIALS AND METHODS

The AAPM TG 119 problem set consists of two preliminary tests to evaluate dose calculating module and four commissioning problems: test prostate, head‐and‐neck (H&N), C‐shaped target, and Multi Target. The first preliminary test is to give 2 Gy at mid‐plane by parallel opposed $10\times 10{\;\text{cm}}^{2}$ field. The second preliminary test is identical except an MLC was used to create bands of 3 cm wide with doses ranging from 0.4 Gy to 2 Gy. Absolute dose measurements with chamber and gamma measurements using Scanditornix, IMRT MatriXX, and OminiPro IMRT software (all IBA Dosimetry, Schwrazenbruck, Germany) are performed. Test prostate structure set consists of prostate GTV, prostate PTV, rectum and bladder. One‐third of rectum is overlapped with prostate PTV. In head‐and‐neck test case with PTV, we have OARs LT & RT parotids and spinal cord. There is 1.5 cm gap between spinal cord and PTV. The C‐shape structure set consists of C‐shape PTV with 1.5 cm inner and 3.7 cm outer radius. OAR core is a cylindrical structure of 1 cm radius and with a gap of 0.5 cm between C‐shape PTV and core. Multi‐target structure set has three cylindrical structures of 4 cm diameter and 4 cm length stacked along the coronal axis. Full description of all the structure sets is available, with dimensions, in AAPM TG 119 report.

**Figure 1:**
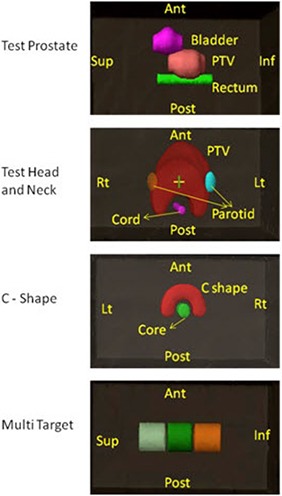
AAPM TG 119 test structure set for prostate, head‐and‐neck, C‐shaped, and Multi Target.

AAPM TG 119 defines the beam arrangement, IMRT goals, and methods for analyzing the dosimetric results. Example plan results are also provided for each test case. Computed tomography (CT) datasets of the test cases were downloaded directly from the AAPM website (www.aapm.org) and imported into our treatment planning system. Figure 1 shows the test structures of these CT's superimposed upon a set of water‐equivalent plastic slabs.

All treatment plans and dose calculations were performed with an Eclipse treatment planning system for a 6 MV beam and a 120 leaf MLC (Eclipse v 8.6.1; AAA 8.6.14, Trilogy with Millennium MLC; Varian Medical Systems, Palo Alto, CA). For each test case, two sets of plans were generated: one with IMRT following the TG 119 guidelines which include orientation and number of gantry angles, and the other with VMAT technique. For test prostate and Multi Target cases, seven static gantry angles 50° apart and one full arc (179.9° to 180.1°) were chosen for IMRT and VMAT plans, respectively. For head‐and‐neck and C‐shaped tests, nine static gantry angles 40° apart for IMRT and two complimentary full arcs for VMAT were used. For all VMAT plans, we maintained the collimator angle at ± 45°, while, for IMRT plans, 0° collimator angle was applied throughout. For all plans, default dose calculation grid size of 2.5 mm was used and heterogeneity corrections were applied. All plans were normalized to an isodose line that ensured coverage of the volume to meet TG 119 requirements. Dose volume constraint and dose prescription for all plans followed the constraints described in TG 119. Tables 1, 2, 3 and 4 shows plan goals given by AAPM TG 119 for test cases prostate, head‐and‐neck, C‐shape, and Multi Target, respectively.

**Table 1:** AAPM TG 119 goals and results with standard deviation (SD), IMRT and VMAT results, and ratio of IMRT and VMAT to AAPM TG 119 data, for test prostate case.

		*TG 119*			*IMRT*	*VMAT*		
*Structure*	*Parameters*	*Goal (Gy)*	*Results (Gy)*	*SD*	*Gy*	*Gy*	*IMRT / TG 119*	*VMAT / TG 119*
PTV	$D_{95}$	>75.60	75.66	0.21	75.67	75.64	1.00	1.00
	$D_{5}$	<83.00	81.43	1.56	81.46	82.30	1.00	1.01
Rectum	$D_{30}$	<70.00	65.36	2.97	54.55	56.12	0.83	0.86
	$D_{10}$	<75.00	73.03	1.50	71.40	72.12	0.98	0.99
Bladder	$D_{30}$	<70.00	43.94	8.78	37.85	31.30	0.86	0.71
	$D_{10}$	<75.00	62.69	8.15	59.44	52.47	0.95	0.84

**Table 2:** AAPM TG 119 goals and results with standard deviation (SD), IMRT and VMAT results, and ratio of IMRT and VMAT to AAPM TG 119 data, for test head‐and‐neck case.

		*TG 119*			*IMRT*	*VMAT*		
*Structure*	*Parameters*	*Goal (Gy)*	*Results (Gy)*	*SD*	*Gy*	*Gy*	*IMRT / TG 119*	*VMAT / TG 119*
PTV	$D_{90}$	50.00	50.28	0.58	50.57	50.00	1.01	0.99
	$D_{99}$	>46.50	47.04	0.52	46.70	48.40	0.99	1.03
	$D_{20}$	<55.00	52.99	0.93	52.16	52.00	0.98	0.98
Cord	Max	<40.00	37.41	2.50	38.34	37.90	1.02	1.01
Parotid	$D_{50}$ (LT)	<20.00	17.98	1.84	19.15	19.25	1.07	1.07
	$D_{50}$(RT)	<20.00	17.98	1.84	18.65	17.98	1.04	1.00

**Table 3:** AAPM TG 119 goals and results with standard deviation (SD), IMRT and VMAT results, and ratio of IMRT and VMAT to AAPM TG 119 data, for C‐shaped case.

		*TG 119*			*IMRT*	*VMAT*		
*Structure*	*Parameters*	*Goal (Gy)*	*Results (Gy)*	*SD*	*Gy*	*Gy*	*IMRT / TG 119*	*VMAT / TG 119*
*PTV*	D	50.00	50.11	0.165	50.00	50.04	1.00	1.00
	$D_{10}$	<55.00	57.02	2.20	54.82	54.93	0.96	0.96
Core	$D_{5}$	<10.00	16.30	3.07	15.85	16.77	0.97	1.03

In this paper, $D_{\text{XX}}$ will refer to the minimum dose that XX% of the volume receives. TG 119 plan comparison parameters are $D_{99}$, $D_{95}$, $D_{90}$, and $D_{5}$ for targets and $D_{50}$, $D_{10}$, $D_{5}$, and $D_{\text{max}}$ for organs at risk (OAR). To score the plans, the following planning comparison tools are used.

1. Conformity index (CI):^(11)^ defined as the ratio between the volume covered by the prescribed isodose and target volume. For test cases prostate, head‐and‐neck, C‐shape, and Multi Target, prescription dose are 75.6 Gy, 50.4 Gy, 50 Gy and 50 Gy, respectively.
2. Homogeneity index (HI):^(5)^ defined as the dose difference normalized to dose prescription $\left(D_{\mathrm{pres}}\right)$ between doses covering 5% ($D_{5}$) and 95% ($D_{95}$) of the PTV.

We also compared total number of monitor units (MU) required for each plan, and the ratio of total number of planned MU of static IMRT and VMAT plans.

## III. RESULTS & DISCUSSION

For preliminary test 1, the measured and calculated point doses at isocenter are 2.01 Gy and 2.0 Gy. For test case 2, they are 1.262 Gy and 1.253 Gy, respectively. For both tests, 95% data points have gamma less than one for the criteria of 3% DD (Dose Difference) and 3 mm DTA (distance to agreement).

**Figure 2:**
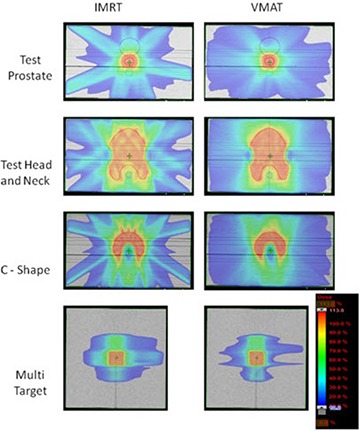
IMRT and VMAT dose distributions for test prostate, head‐and‐neck, C‐shaped, and Multi Target.

Figure 2 shows axial plane dose distributions of IMRT and VMAT plans of test prostate, test head‐and‐neck, and C‐shaped. And frontal plane dose distributions of IMRT and VMAT plans of Multi Target cases.

Table 1 shows the test prostate results. In addition to the dosimetric endpoints required by AAPM TG 119, we also provide a ratio between our planning results and the benchmark values of TG 119. PTV $D_{95}$ and $D_{5}$ of IMRT and VMAT plans are comparable to AAPM TG 119 plans, wherein the dose prescription is 75.6 Gy to $D_{95}$. All criteria meet or exceed the requirements of TG 119. (Figure 3a) shows test prostate case IMRT and VMAT DVHs for PTV, rectum and bladder. IMRT and VMAT plans have comparable DVH.

**Figure 3(a):**
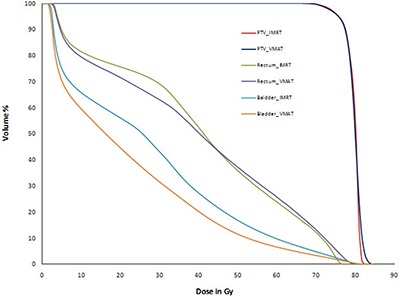
Test prostate plan comparison DVH.

Table 2 shows the same results as Table 1, but for test head‐and‐neck plans prescribed to receive 50 Gy to PTV $D_{90}$. Both IMRT and VMAT plans PTV coverage ($D_{99}$) are comparable to AAPM TG 119 results. VMAT's $D_{99}$ has 4% and 3% better coverage compared to IMRT and TG 119 results, respectively. For both VMAT and IMRT plans, the PTV $D_{20}$ is 2% less than AAPM TG 119 mean dose and well within the planning goal. The maximum cord dose of IMRT and VMAT were 38.34 Gy and 37.90 Gy, respectively, and they are below the constraint (< 38.50 Gy). While the dose constraint for parotid is to have $D_{50}$ less than 20 Gy, IMRT and

VMAT $D_{50}$ doses are maximum 1.17 Gy and 1.27 Gy higher than AAPM TG 119 $D_{50}$ mean dose 17.98 Gy, respectively. But AAPM TG 119 parotid $D_{50}$ standard deviation is 1.84 Gy and our results are within one standard deviation. (Figure 3b) shows test head‐and‐neck IMRT and VMAT dose‐volume histograms (DVH) for PTV, cord, left, and right parotids. In case of test head‐and‐neck, IMRT and VMAT dose volume histograms are comparable.

Table 3 shows IMRT and VMAT results for a test case C‐shaped. Here PTV plan prescription is 50 Gy to outer target. Both IMRT and VMAT plans achieved PTV $D_{10}$ very close to the planning goal of 55 Gy. Both plans fail to meet the $D_{5}$ hard constraint of OAR core, but the plan results are comparable to TG 119 plan results. AAPM TG 119 core $D_{5}$ mean and standard deviation are 16.03 Gy and 3.07 Gy. Our IMRT and VMAT $D_{5}$ doses are within one standard deviation of AAPM TG 119 $D_{5}$ mean dose. (Figure 3c) shows test case C‐shaped plan DVHs for IMRT and VMAT plans. IMRT and VMAT core and target DVHs are comparable.

**Figure 3(b):**
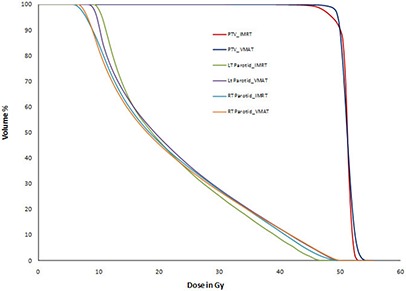
Test head‐and‐neck plan comparison DVH.

For Multi Target test case, VMAT and IMRT plans achieved the planning goals. When compared to the benchmark TG 119 results, our IMRT and VMAT plans have more homogenous coverage to superior and inferior targets and similar results for the center target. IMRT and VMAT plans have 6% and 3% more homogenous coverage for superior and inferior targets, respectively. Table 4 shows the plan comparison results for Multi Target and our plan results compared with AAPM TG 119 results. (Figure 3d) shows Multi Target plan comparison DVHs of IMRT and VMAT plans. IMRT and VMAT dose volume histograms of superior, inferior, and center target are comparable.

**Figure 3(c):**
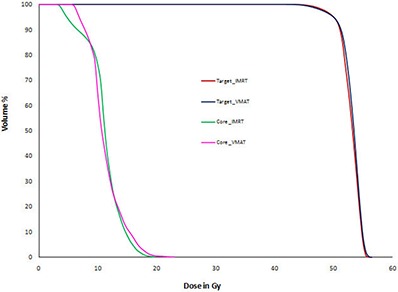
C‐shaped plan comparison DVH.

**Figure 3(d):**
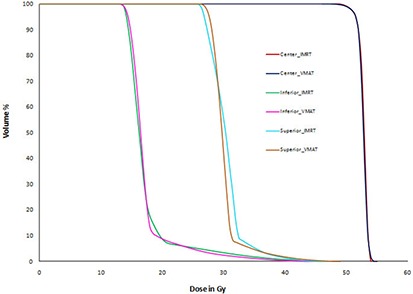
Multi Target plan comparison DVH.

**Table 4:** AAPM TG 119 goals and results with standard deviation (SD), IMRT and VMAT results, and ratio of IMRT and VMAT to AAPM TG 119 data, for Multi Target case.

		*TG 119*			*IMRT*	*VMAT*		
*Structure*	*Parameters*	*Goal (Gy)*	*Results (Gy)*	*SD*	*Gy*	*Gy*	*IMRT / TG 119*	*VMAT / TG 119*
Center	$D_{99}$	>50.00	49.55	1.62	50.07	50.00	1.01	1.01
	$D_{10}$	<53.00	54.55	1.73	53.58	53.52	0.98	0.98
Superior	$D_{99}$	>25.00	25.16	0.85	26.21	26.87	1.04	1.07
	$D_{10}$	<35.00	34.12	3.04	32.43	31.33	0.95	0.92
Inferior	$D_{99}$	>12.50	14.07	1.85	13.64	13.67	0.97	0.97
	$D_{10}$	<25.00	24.18	2.72	19.60	19.04	0.81	0.79

Table 5 shows plan evaluation results of test cases test prostate, test head‐and‐neck, C‐shaped, and Multi Target. It also shows the planned monitor units (MU) of all plans and the ratio of MUs for IMRT and VMAT plans. The conformity index (CI) of all four cases is comparable. Only for test case C‐shaped, IMRT plan conformity index 1.18 is higher when compared to VMAT plan CI of 1.08. For each test case, the HI of IMRT and VMAT plans are essentially identical (within 1%). Except for C‐shaped case, all three plans have HI less than 10%. HI increased as the constraints for the OAR become harder.

**Table 5:** Plan evaluation results.

	*Test Prostate*		*Test H&N*		*C‐Shaped*		*Multi Target*	
*Case*	*IMRT*	*VMAT*	*IMRT*	*VMAT*	*IMRT*	*VMAT*	*IMRT*	*VMAT*
Conformity Index (CI)	1.05	1.04	1.10	1.09	1.18	1.08	1.23	1.23
Homogeneity Index (%)	7.66	8.8	7.22	6.57	11.02	10.54	4.56	4.58
MU	590	529	1251	621	1401	729	821	476
MU Ratio	1.11	1	2.01	1	1.92	1	1.72	1

IMRT and VMAT plans showed similar and comparable results for all four test cases. Table 5 also has the information about treatment planning MU of all plans and the ratio of IMRT and VMAT plans monitor units (MU). IMRT plans have total planned MU as 590, 1251, 1401, and 821 for test prostate, test head‐and‐neck, C‐shape, and Multi Target cases, respectively. VMAT plans have total planned MU as 529, 621, 729, and 476 for the four cases, respectively. IMRT verses VMAT plans total planned MU ratios are 1.11, 2.01, 1.8, and 1.72, respectively. As the complexity of plan increases, there is an increase in the total number of MU required for an IMRT plan and VMAT plan. However, the increase in the planned MU with complexity is less for VMAT plans compared to IMRT plans. To produce comparable distribution for complex cases like test head‐and‐neck and C‐shaped, IMRT required almost double the number of MU required by VMAT plan. This is likely due to greater degrees of freedom provided by VMAT. For VMAT plans, apart from the reduction of MU required, number of beam “MODE UPs” also reduced to one or two times when compared 7 to 9 times of IMRT plan. On average, it takes 30 sec to mode up linear accelerator. So, there is another additional two to three minutes reduction in treatment time due to fewer beams “MODE UP” with VMAT plan. The VMAT optimization time is considerably high compared to IMRT optimization.

Table 6 shows IMRT and VMAT plan's gamma analysis and point dose results measured using Scanditornix, IMRT MatriXX and OminiPro IMRT software. The ratio of measured and calculated point doses are also shown there. All gamma evaluation results show gamma less than one for more than 95% data points with the criteria of 3% DD (dose difference) and 3 mm DTA (distance to agreement). The point dose results are within 2% of planned dose values.

**Table 6:** Gamma analysis and point dose results of IMRT and VMAT plans of AAPM TG 119 test cases. Gamma criteria of 3% dose difference (DD) and 3 mm distance to agreement (DTA).

	*VMAT*				*IMRT*			
	*Point Dose (Gy)*			*Gamma*	*Point Dose (Gy)*			*Gamma <1(%)*
*Test Case*	*Measured*	*Planned*	*ratio*	<*1(%)*	*Measured*	*Planned*	*ratio*	<*1(%)*
Prostate	1.989	2.066	0.98	96.79	2.0	2.0072	0.996	97.67
Head & Neck	1.915	1.943	1.014	98.15	1.960	1.984	0.988	95.92
C‐shaped	2.263	2.292	0.987	96.86	2.301	2.325	0.987	96.11
Multi Target	2.301	2.280	1.009	98.99	2.245	2.288	0.981	97.92

## IV. CONCLUSIONS

TG 119 test case suite is very useful to understand and compare different optimization modules. The various levels of complexity in the test cases explore the new optimization module capability against established optimization module. TG 119 goals help to establish a baseline and benchmark commissioning data for IMRT. They are also helpful to gain confidence in new modalities like VMAT and to test its capabilities at preclinical implementation stage. True benchmarking of VMAT programs, as is done in TG 119, would require a multi‐institutional and multiple‐vendor studies.

## References

[1] Ling CC , Burman C , Chui CS , et al. Conformal radiation treatment of prostate cancer using inversely‐planned intensity‐modulated photon beams produced with dynamic multileaf collimation. Int J Radiat Oncol Biol Phys. 1996;35(4):721–30. 8690638 10.1016/0360-3016(96)00174-5

[2] Yu CX . Intensity‐modulated arc therapy with dynamic multileaf collimation: an alternative to tomotherapy. Phys Med Biol. 1995;40(9):1435–49. 8532757 10.1088/0031-9155/40/9/004

[3] Otto K . Volumetric modulated arc therapy: IMRT in a single gantry arc. Med Phys. 2008;35(1):310–17. 18293586 10.1118/1.2818738

[4] Cozzi L , Dinshaw KA , Shrivastava SK , et al. A treatment planning study comparing volumetric arc modulation with RapidArc and fixed field IMRT for cervix uteri radiotherapy. Radiother Oncol. 2008;89(2):180–91. 18692929 10.1016/j.radonc.2008.06.013

[5] Clivio A , Fogliata A , Franzetti‐Pellanda A , et al. Volumetric‐modulated arc radiotherapy for carcinomas of the anal canal: a treatment planning comparison with fixed field IMRT. Radiother Oncol. 2009;92(1):118–24. 19181409 10.1016/j.radonc.2008.12.020

[6] Shaffer R , Nichol AM , Vollans E , et al. A comparison of volumetric modulated arc therapy and conventional intensity‐modulated radiotherapy for frontal and temporal high‐grade gliomas. Int J Radiat Oncol Biol Phys. 2010;76(4):1177–84. 19560880 10.1016/j.ijrobp.2009.03.013

[7] Ling CC , Zhang P , Archambault Y , Bocanek J , Tang G , Losasso T . Commissioning and quality assurance of RapidArc radiotherapy delivery system. Int J Radiat Oncol Biol Phys. 2008;72(2):575–81. 18793960 10.1016/j.ijrobp.2008.05.060

[8] LoSasso T , Chui CS , Ling CC . Comprehensive quality assurance for the delivery of intensity modulated radiotherapy with a multileaf collimator used in the dynamic mode. Med Phys. 2001;28(11):2209–19. 11764024 10.1118/1.1410123

[9] Ezzell GA , Galvin JM , Low D , et al. Guidance document on delivery, treatment planning, and clinical implementation of IMRT: report of the IMRT Subcommittee of the AAPM Radiation Therapy Committee. Med Phys. 2003;30(8):2089–115. 12945975 10.1118/1.1591194

[10] Ezzell GA , Burmeister JW , Dogan N , et al. IMRT commissioning: multiple institution planning and dosimetry comparisons, a report from AAPM Task Group 119. Med Phys. 2009;36(11):5359–73. 19994544 10.1118/1.3238104

[11] Feuvret L , Noel G , Mazeron JJ , Bey P . Conformity index: a review. Int J Radiat Oncol Biol Phys. 2006;64(2):333–42. 16414369 10.1016/j.ijrobp.2005.09.028

